# Osteoclast differentiation and dynamic mRNA expression during mice embryonic palatal bone development

**DOI:** 10.1038/s41598-023-42423-4

**Published:** 2023-09-13

**Authors:** Yongzhen Lai, Yan Guo, Caiyu Liao, Chuanqing Mao, Jing Liu, Chengyan Ren, Wen Yang, Lin Luo, Weihui Chen

**Affiliations:** 1 Department of Oral and Craniomaxillofacial Science, Fujian Medical University Union Hospital, No. 28, Xinquan Road, Fuzhou, 350001 Fujian China; 2 Stomatological Key Laboratory of Fujian College and University, Fuzhou, China; 3 Department of Stomatology, Fujian Maternal and Child Health Hospital, No. 18 Dao Shan Road, Fuzhou, 350001 Fujian China

**Keywords:** Bone development, Bone remodelling, Cell growth, Embryogenesis, Embryology

## Abstract

This study is the first to investigate the process of osteoclast (OCL) differentiation, its potential functions, and the associated mRNA and signalling pathways in embryonic palatal bone. Our findings suggest that OCLs are involved in bone remodelling, bone marrow cavity formation, and blood vessel formation in embryonic palatal bone. We observed TRAP-positive OCLs at embryonic day 16.5 (E16.5), E17.5, and E18.5 at the palatal process of the palate (PPP) and posterior and anterior parts of the palatal process of the maxilla (PPMXP and PPMXA, respectively), with OCL differentiation starting 2 days prior to TRAP positivity. By comparing the key periods of OCL differentiation between PPMX and PPP (E14.5, E15.5, and E16.5) using RNA-seq data of the palates, we found that the PI3K-AKT and MAPK signalling pathways were sequentially enriched, which may play critical roles in OCL survival and differentiation. Csf1r, Tnfrsff11a, Ctsk, Fos, Tyrobp, Fcgr3, and Spi1 were significantly upregulated, while Pik3r3, Tgfbr1, and Mapk3k7 were significantly downregulated, in both PPMX and PPP. Interestingly, Tnfrsff11b was upregulated in PPMX but downregulated in PPP, which may regulate the timing of OCL appearance. These results contribute to the limited knowledge regarding mRNA-specific steps in OCL differentiation in the embryonic palatal bone.

## Introduction

In mammals, the palate consists anteriorly of the bony hard palate and posteriorly of the muscular soft palate. The hard palate separates the mouth and nasal cavity to ensure normal swallowing, speech and hearing functions and provides the attachment point of the soft palate^1,2^. The secondary palate-derived hard palate consists anteriorly of the palatal processes of the maxilla (PPMX) and posteriorly of the palatal processes of the palatine (PPP), whereas the primary palate consists of the small triangular piece of bone extending from anterior of the palatal processes of the maxilla to the alveolar bone of the incisors^3^. Defects in palatal bone formation result in submucous cleft palate (SMCP). Despite the high frequency of SMCP in humans, only recently have a few mouse models of this congenital anomaly begun to provide insight into bone formation in the secondary palate^4,5^. These limited palatal bone defect models established in transgenic mice helped elucidate the roles of Tbx22, Bmpr1a, SHOX2, and FGF8 in the transformation of palatal mesenchymal cells into osteoblasts^3,6–8^. In these studies, distinct gene expression patterns along the antero-posterior axis of the secondary palate were generally recognized. For further research, we described morphogenesis and heterogeneity in the osteogenic and angiogenic gene profiles of the PPMX and PPP in a previous study^9^.

Palatal bone formation occurs by intramembranous ossification. Intramembranous osteogenesis is mainly determined by a series of consecutive and overlapping processes, such as the osteogenic fate of mesenchymal pluripotent stem cells, the differentiation and maturation of osteoblasts, the secretion and mineralization of extracellular matrix (ECM), the differentiation and bone remodelling of osteoclasts (OCLs), and the generation of blood vessels^10,11^. We previously reported for the first time that OCLs in the embryonic palate had morphological differences between the anterior and posterior axes of the palate^9^. OCLs are multinucleated cells belonging to the myeloid lineage with resorptive abilities and are generated via the fusion of monocytes/macrophage precursor cells, forming the bone marrow cavity during development^12,13^. Many studies have proven the heterogeneity of OCLs in different types of bone^14–17^. Research on embryonic OCLs mainly focuses on endochondral osteogenesis, while reports on intramembranous osteogenesis are rare. At present, some sporadic studies on intramembranous osteoblasts and OCLs in the embryonic stage suggest that OCLs may play a role in promoting bone matrix maturation and determining bone morphology^18–20^. However, the differentiation process of embryonic secondary palatal bone (PPMX and PPP) OCLs and their relationship to intramembranous osteogenesis remain unclear.

The main signalling pathways that drive osteoclastogenesis show that binding of the CSF1R receptor by its ligands (typically CSF1) activates the Grb2-ERK and the Src-PI3K-AKT pathways, which promote cell survival and proliferation^21,22^. RANKL binds its decoy receptor osteoprotegerin (OPG)^23^ or its cell-surface receptor RANK. RANKL-bound RANK recruits several tumour necrosis factor receptor–associated factor (TRAF) molecules, mainly TRAF6, which activates the IKKNFkB and Src-PI3K-AKT pathways and activates MAPKs. Collectively, these signalling events induce the expression of key transcription factors, including NFATc1, Fos, and Jun, that drive the expression of downstream genes, including Acp5 (encodes TRAP), Ctsk (Cathepsin K), Dcstamp (DC-STAMP), Mmp9 (Matrix Metalloproteinase 9), Atp6v0d2 (the D2 subunit of the V0 complex of the V-ATPase), and Nfatc1 itself^24^. NFATc1 is activated by the phosphatase calcineurin and by calcium/calmodulin-dependent protein kinase IV (CaMK IV), whose activation requires calcium oscillations induced by the ITAM-containing proteins DAP12 or FCRy^25–28^. However, the molecular regulation of OCL differentiation in embryonic palatal bone (PPMX and PPP) remains unclear.

The development of embryonic palatal bone has attracted increasing attention. However, the differentiation of OCLs in this process has not been reported. Therefore, the aim of this study was to elucidate the differentiation process of OCLs and its relationship with mineralization and angiogenesis during embryonic palatal bone development and to identify the signalling pathways and mRNA dynamic expression associated with OCLs.

## Methods

This study was approved by the Biomedical Ethics Committee of Fujian Medical University (FJMU IACUC 2020-0026). Animal experiments were carried out in compliance with the ARRIVE guidelines (http://www.nc3rs.org.uk/page.asp?id=1357) and the NIH guidelines (Guide for the Care and Use of Laboratory Animals).

### Animals

ICR mice, 10–12 weeks of age, were purchased from the Shanghai Laboratory Animal Center, China (licence no. SCXK 2012–0002). Three pairs of ICR mice were mated, and embryonic day 0.5 (E0.5) was defined as the day of vaginal plug discovery.

**Figure 1:**
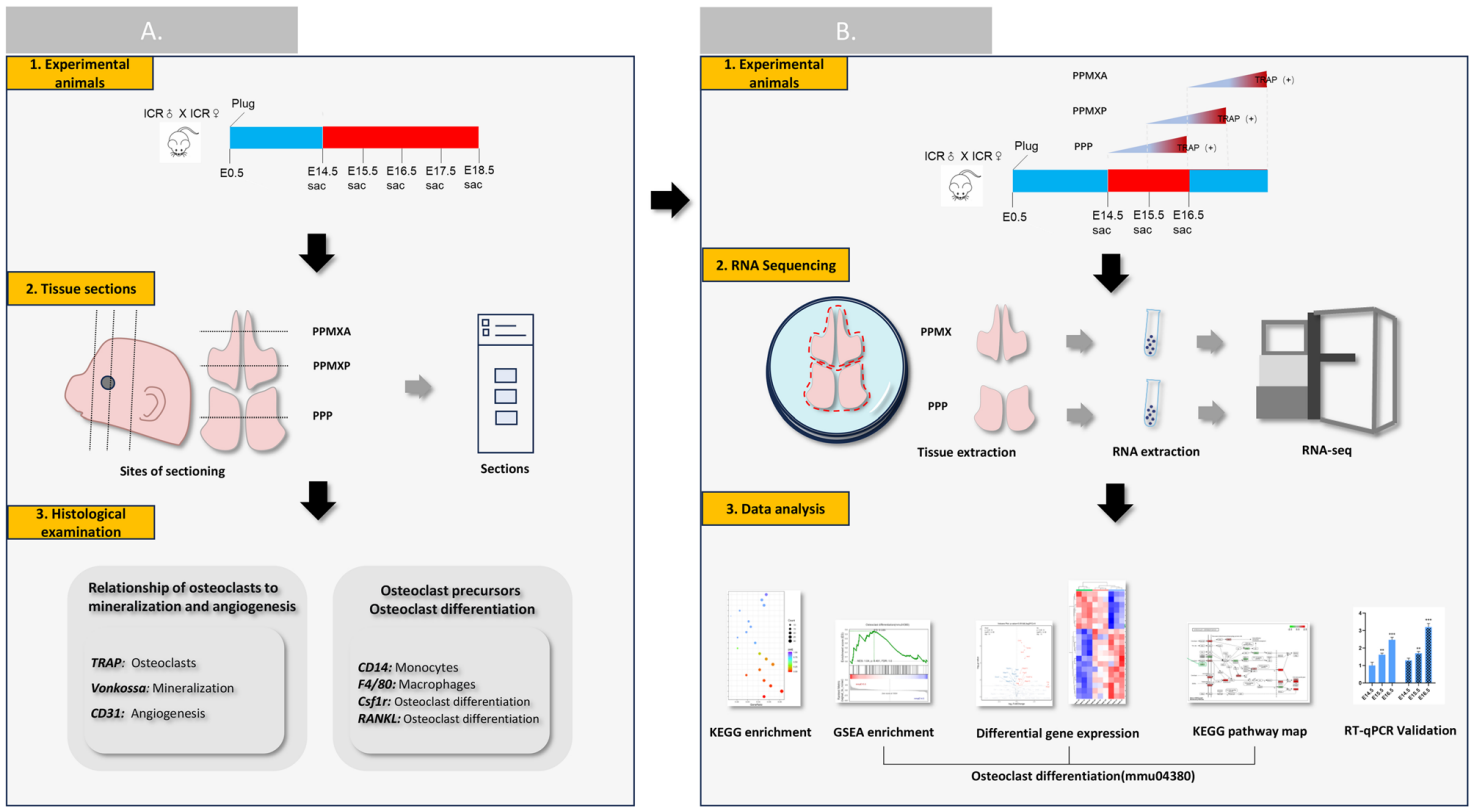
Study workflow. (**A**) Osteoclast-related histological and immunohistochemical findings in embryonic palatal bone. (**B**) Osteoclast-related RNA sequencing and data analysis in embryonic palatal bone.

At the embryonic developmental stages E14.5, E15.5, E16.5, E17.5, and E18.5, three pregnant mice were euthanized per time point. From each of these pregnant mice, three embryos were selected for subsequent histological and immunofluorescence staining. Additional pregnant mice were euthanized at E14.5, E15.5, and E16.5. From each group of three pregnant mice, embryos were harvested for RNA sequencing (RNA-seq) and qRT-PCR analyses (Fig. 1).

### Histology and immunohistochemistry

All embryos were fixed overnight in 4% paraformaldehyde, dehydrated through graded alcohols, embedded in paraffin, and sectioned at a thickness of 4 µm. For TRAP staining, sections were incubated with pure water at 37 °C for 2 h, placed in the prepared TRAP staining solution at 37 °C for dark staining for 1 h, and then counterstained with haematoxylin. For von Kossa staining, the sections were dropped with the prepared von Kossa silver staining solution and exposed to bright light for 15 min, followed by Hywave solution for 2 min, and finally counterstained with eosin. A TRAP kit (387A-1KT, Sigma, USA) and von Kossa kit (G3282, Solarbio, China) were used as described in our previous study^9^. For immunohistochemistry, sections were subjected to antigen retrieval and then blocked with normal goat serum, followed by incubation overnight at 4 °C with the primary antibodies anti-CD14 (1:200, ab181470, Abcam), anti-F4/80 (1:200, ab300421, Abcam), anti-CSF1R (1:200, ab254357, Abcam), and anti-RANKL (1:200, ab45039, Abcam). Anti-CD31 (1:300, ab28364, Abcam) and goat anti-rabbit IgG H&L (1:200, ab150080, Abcam) were used.

### Tissue separation and RNA-sequencing

E14.5, E15.5, E16.5 pregnant mice were euthanized by decapitation and soaked in 100% alcohol for one minute. Afterwards, embryos were obtained and transferred to ice-cold Hanks liquid. The mandibles and skulls of the embryos were removed. The PPMX and PPP of E14.5–E16.5 embryos (n = 3 for each group) were dissected out under a stereomicroscope and then stored in RNAlater (BI, Israel) for RNA-seq. After RNA quantification and qualification, a total amount of 1 μg RNA per sample was used as input material for the RNA sample preparations. Sequencing libraries were generated using NEBNext® UltraTM RNA Library Prep Kit for Illumina® (NEB, USA) following manufacturer’s recommendations and index codes were added to attribute sequences to each sample. Generation System using TruSeq PE Cluster Kit v3-cBot-HS (Illumia) according to the manufacturer’s instructions. After cluster generation, the library preparations were sequenced on an Illumina Novaseq platform and 150 bp paired-end reads were generated. RNA-seq data were shared with our previous study^9^. Data were deposited in the Gene Expression Omnibus database (https://www.ncbi.nlm.nih.gov/geo/query/acc.cgi?acc=GSE240930, GSE240930).

### Bioinformatic analysis

FeatureCounts v1.5.0-p3 was used to count the reads numbers mapped to each gene. And then FPKM of each gene was calculated based on the length of the gene and reads count mapped to this gene. Prior to differential gene expression analysis, for each sequenced library, the read counts were adjusted by edgeR program package through one scaling normalized factor. Differential expression analysis of two conditions was performed using the edgeR R package (3.18.1)**.** Expression patterns of genes expression from E14.5 to E16.5 in PPP and PPMX were compared. The log2 (fold-change cut-off) was set at greater than 0 for analyses of differential expression and the Q-value was set to less than 0.05 to find differentially expressed genes (DEGs). The E15.5 vs. E14.5 pairwise, E16.5 vs. E15.5 pairwise, and E16.5 vs. E14.5 pairwise comparisons with the E14.5 group, E15.5 group, and E14.5 were used as the control groups, respectively.

Kyoto Encyclopedia of Genes and Genomes (KEGG) pathway analysis were conducted by using clusterProfiler6 to reveal the unique biological significance and key pathways associated with DEGs.We performed KEGG enrichment analysis of the total DEGs (Criteria: p-value < 0.05, significantly enriched). GSEA enrichment analysis of pathways related to osteoclast differentiation (mmu04380) was performed (Criteria: |NES|> 1, p-val < 0.05, FDR q-val < 0.25, significantly enriched).The heatmap and volcano map of DEGs related to osteoclast differentiation (mmu04380) are displayed, and the corresponding KEGG pathway map is depicted.

### RNA collection and Quantitative real-time polymerase chain reaction (qRT-PCR)

RNA was purified from the PPMX and PPP of E14.5, E15.5 and E16.5 embryos (n = 3 for each group) using TRIzol reagent (Invitrogen, Carlsbad, CA). Five hundred nanograms of total RNA was submitted for reverse transcription using PrimeScript RT Master Mix (TaKaRa Bio, Inc.), incubation at 37 °C for 15 min and then 85 °C for 5 s to terminate the reaction, with a final volume of 20 μL. The qPCR cycle was conducted using SYBR Premix Ex Taq II (Perfect Real Time; TaKaRa, Bio, Inc.) on an Applied Biosystems 7500 real-time PCR machine (Life Technologies) using 2 µL of the cDNA obtained in the RT reaction. As shown in Table 1, primers were designed using the NCBI primer design tool (Primer-BLAST). The PCR program was 2 min at 95 °C for predenaturation, 5 s at 95 °C and 40 s at 60 °C for forty cycles. For each gene, PCR was carried out in triplicate, and the relative levels of mRNAs were normalized to that of GAPDH expression and then normalized to PPMXA-E14.5 samples. Amplification efficiency was evaluated using standard curve analysis. Statistical tests were performed using the SPSS 22.0 program, and a P value less than 0.05 was considered statistically significant.

**Table 1:** DNA sequences of the primers used in this study.

Name	DNA sequences of primers	Amplification efficiency (%)
Csf1r	Forward: TGTCATCGAGCCTAGTGGC Reverse: GGTCCAAGGTCCAGTAGGG	101.20
Tnfrsf11	Forward: AGCCGAGACTACGGCAAGTA Reverse: AAAGTACAGGAACAGAGCGATG	98.20
Tnfrsf11a	Forward: CCAGGAGAGGCATTATGAGCA Reverse: ACTGTCGGAGGTAGGAGTGC	94.40
Tnfrsf11b	Forward: CCTTGCCCTGACCACTCTTAT Reverse: CACACACTCGGTTGTGGGT	102.30
Ctsk	Forward: CTCGGCGTTTAATTTGGGAGA Reverse: TCGAGAGGGAGGTATTCTGAGT	99.60
Acp5	Forward: CACTCCCACCCTGAGATTTGT Reverse: CCCCAGAGACATGATGAAGTCA	98.70
Pik3ca	Forward: CCACGACCATCTTCGGGTG Reverse: GGGGAGTAAACATTCCACTAGGA	93.50
Akt1	Forward: ATGAACGACGTAGCCATTGTG Reverse: TTGTAGCCAATAAAGGTGCCAT	104.30
Mapk3	Forward: TCCGCCATGAGAATGTTATAGGC Reverse: GGTGGTGTTGATAAGCAGATTGG	95.30
Mapk14	Forward: TGACCCTTATGACCAGTCCTTT Reverse: GTCAGGCTCTTCCACTCATCTAT	95.10

### Ethics approval

This study was approved by the Biomedical Ethics Committee of Fujian Medical University (FJMU IACUC 2020-0026).

## Results

### Differentiation of OCLs in embryonic palatal bone and its relationship with intramembrane osteogenesis

#### TRAP-positive OCLs were found at PPMX-A at E18.5

**Figure 2:**
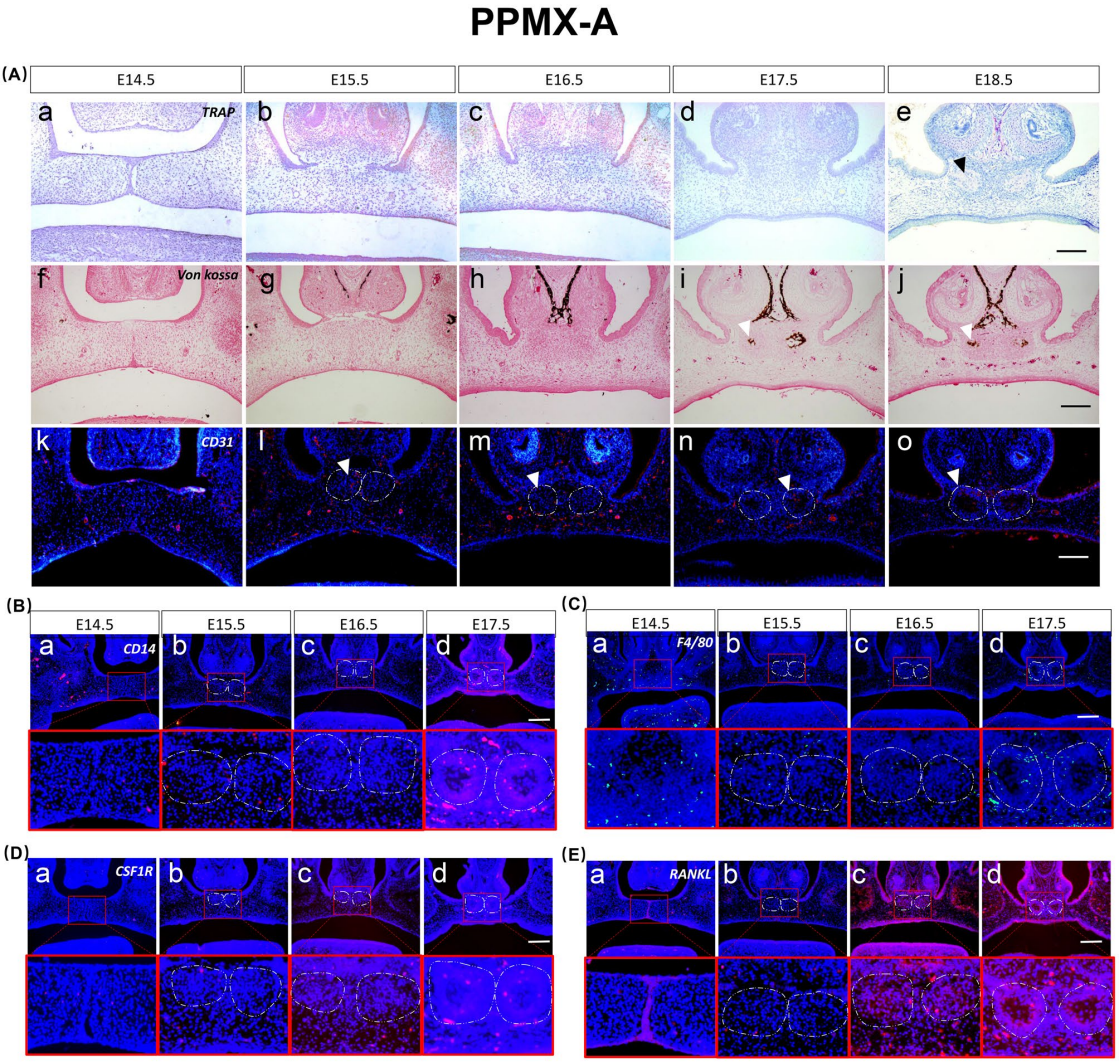
Osteoclast differentiation process of the palatal bone (PPMX-A). (**A**) TRAP staining from E14.5 to E18.5 “a–e”, von Kossa staining from E14.5 to E18.5 “f–j”. Immunofluorescence staining expression patterns of CD31 from E14.5 to E18.5 “k–o”. (**B**–**E**) Immunofluorescence staining expression patterns of CD14, F4/80, CSF1R and RANKL during palatal osteoclastogenesis from E14.5 to E17.5. Black triangles represent TRAP-positive regions, and white triangles represent mineralizing and CD31-positive regions. Scale bar 500 µm.

At E14.5, CD31-, CD14-, CSF1R-, F4/80-, and RANKL-positive cells were few and scattered in the surrounding loose mesenchyme (Fig. 2A.a.k.f,B.a–E.a).

At E15.5, mesenchymal condensation was observed, CD31-positive cells invaded, and scattered CD14-, F4/80-, and CSF1R-positive cells were detected. No RANKL-positive cells were detected (Fig. 2A.b.g.l,B.b–E.b).

At E16.5, further mesenchymal condensation and low levels of scattered CD31-, CD14-, and F4/80-positive cells were observed, but CSF1R- and RANKL-positive expression increased (Fig. 2A.c.h.m,B.c–E.c).

At E17.5, calcification occurred, and CD31-, CD14-, and F4/80-positive expression was enhanced. CSF1R- and RANKL-positive cells were highly expressed (Fig. 2A.d.i,n,B.d–E.d).

At E18.5, TRAP was expressed in the nasal side of the condensed mesenchyme, and CD31-positive expression was enhanced at this site (Fig. 2A.e,j,o).

#### TRAP-positive OCLs were found at PPMX-P at E17.5

**Figure 3:**
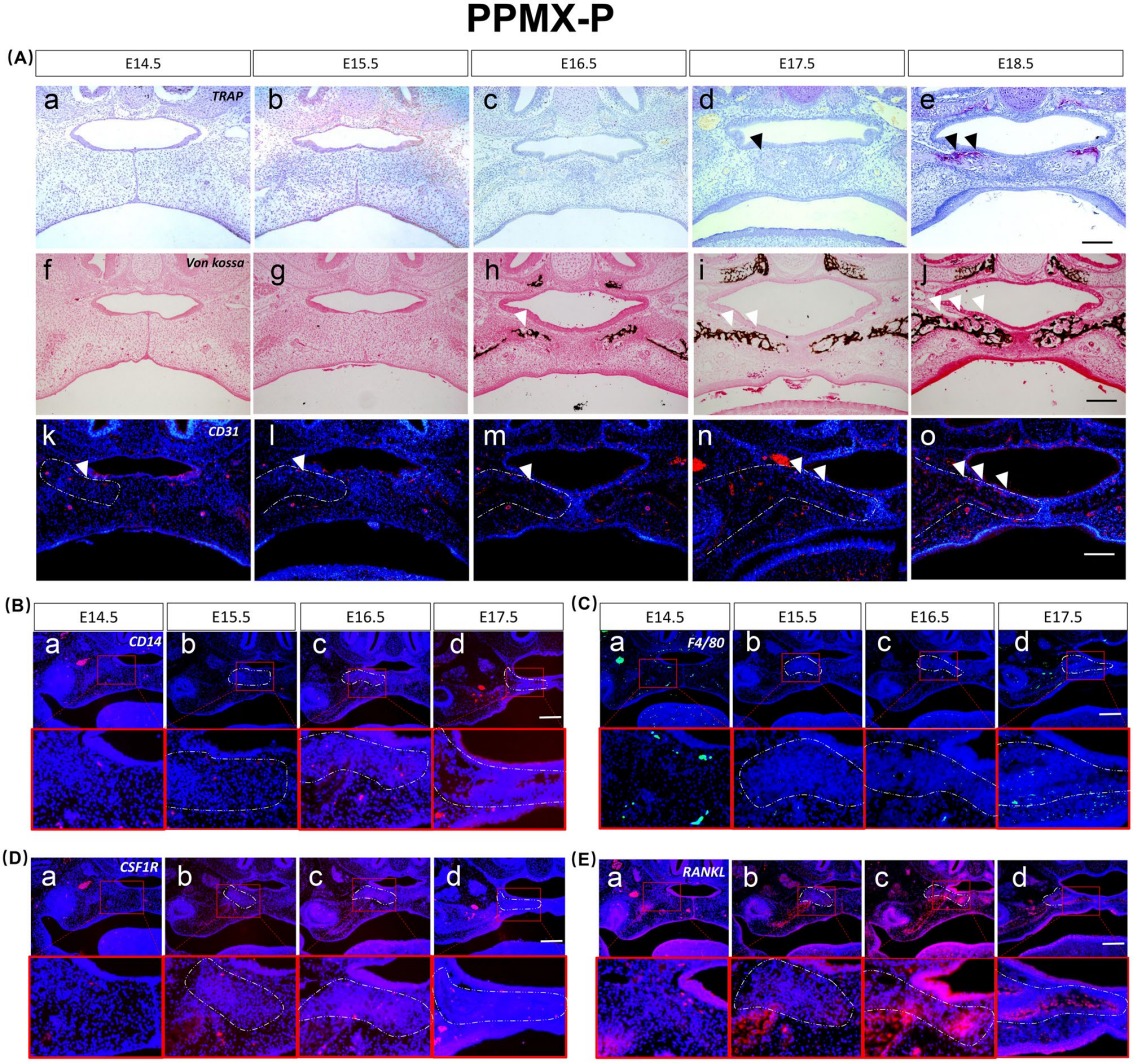
Osteoclast differentiation process of the palatal bone (PPMX-P). (**A**) TRAP staining from E14.5 to E18.5 “a–e”, von Kossa staining from E14.5 to E18.5 “f–j”. Immunofluorescence staining expression patterns of CD31 from E14.5 to E18.5 “k–o”. (**B**–**E**) Immunofluorescence staining expression patterns of CD14, F4/80, CSF1R and RANKL during palatal osteoclastogenesis from E14.5 to E17.5. Black triangles represent TRAP-positive regions, and white triangles represent mineralizing and CD31-positive regions. Scale bar 500 µm.

At E14.5, mesenchymal condensation was observed. Few and scattered CD31-, CD14-, CSF1R-, F4/80-, and RANKL-positive cells were observed on the nasal side (Fig. 3A.a.f.k,B.a–E.a).

At E15.5, further mesenchymal condensation occurred, CD31-, CD14-, and F4/80-positive cells were still scattered in small amounts, and CSF1R- and RANKL-positive expression increased (Fig. 3A.b.g.l,B.b–E.b).

At E16.5, calcification occurred, and CD31-, CD14-, and F4/80-positive expression was enhanced. CSF1R- and RANKL-positive cells were highly expressed (Fig. 3A.c.h.m,B.c–E.c).

At E17.5, TRAP was expressed in the nasal side of the condensed mesenchyme, with enhanced positive expression of CD31 and CD14, and F4/80 was slightly expressed in the osteoclast formation area, while it was enhanced in other areas. The expression range of RANKL and CSF1R was reduced and concentrated in the medial bone marrow cavity (Fig. 3A.d.i.n,B.d–E.d).

At E18.5, TRAP expression was enhanced, and the distribution expanded. The bone marrow cavity was enlarged in this region. CD31 expression was also enhanced (Fig. 3A.e,j,o).

#### TRAP-positive OCLs were found at PPP at E16.5

**Figure 4:**
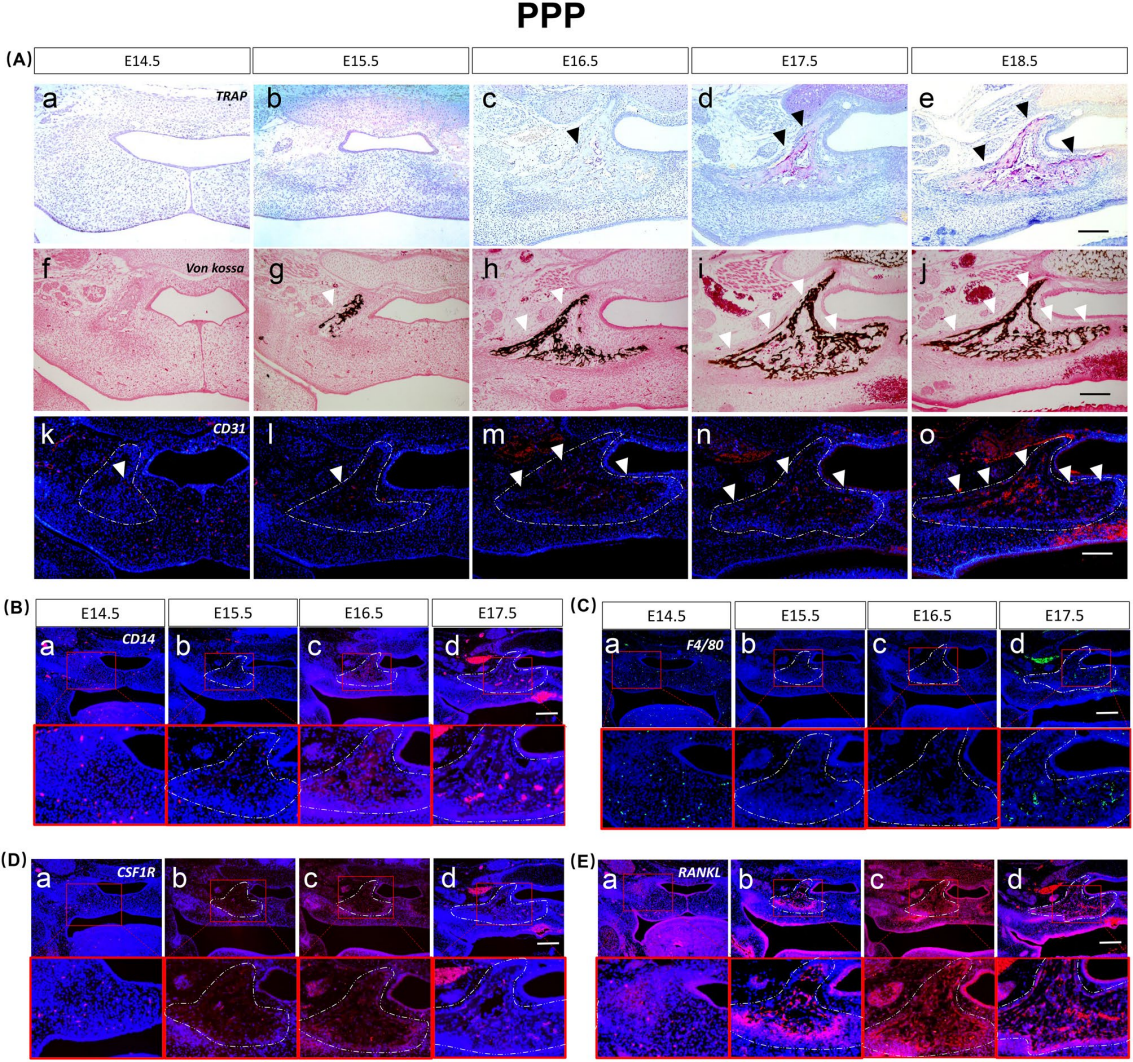
Osteoclast differentiation process of the palatal bone (PPP). (**A**) TRAP staining from E14.5 to E18.5 “a–e”, von Kossa staining from E14.5 to E18.5 “f–j”. Immunofluorescence staining expression patterns of CD31 from E14.5 to E18.5 “k–o”. (**B**–**E**) Immunofluorescence staining expression patterns of CD14, F4/80, CSF1R and RANKL during palatal osteoclastogenesis from E14.5 to E17.5. Black triangles represent TRAP-positive regions, and white triangles represent mineralizing and CD31-positive regions. Scale bar 500 µm.

At E14.5, mesenchymal condensation and unmineralized bone appeared in the vertical ramus. CD31-, CD14-, CSF1R-, F4/80-, and RANKL-positive cells were observed in this area (Fig. 4A.a.i.k,B.a–E.a).

At E15.5, calcification occurred in the vertical ramus, and CD31-, CD14-, and F4/80-positive expression was low and scattered. CSF1R- and RANKL-positive cells were highly expressed (Fig. 4A.b.g.l,B.b–E.b).

At E16.5, TRAP was expressed in the vertical ramus, with enhanced positive expression of CD31 and CD14, and F4/80 was slightly expressed in the osteoclast formation area, while it was enhanced in other areas. CSF1R- and RANKL-positive cells were highly expressed (Fig. 4A.c.h.m,B.c–E.c).

At E17.5, TRAP expression was enhanced, and the distribution expanded. The bone marrow cavity was enlarged in this region. CD31, CD14, and F4/80 expression was also enhanced. The expression range of RANKL and CSF1R was reduced and concentrated in the medial bone marrow cavity (Fig. 4A.d.i.n,B.d–E.d).

At E18.5, TRAP expression was enhanced, and the distribution expanded to the horizontal ramus. The bone marrow cavity was enlarged in this region. CD31 expression was also enhanced (Fig. 4A.e,j,o).

### Expression patterns of osteoclast-related genes and signalling in embryonic palatal bone

The PCA plot and Fragments Per Kilobase of exon model per Million mapped fragments (FPKM) density distribution show variability in gene expression among biological replicates between PPMX and PPP at E14.5, E15.5 and E16.5 (Supplementary Fig. S1A,B).

#### The osteoclast differentiation pathway of PPMX was upregulated at E16.5–E15.5

**Figure 5:**
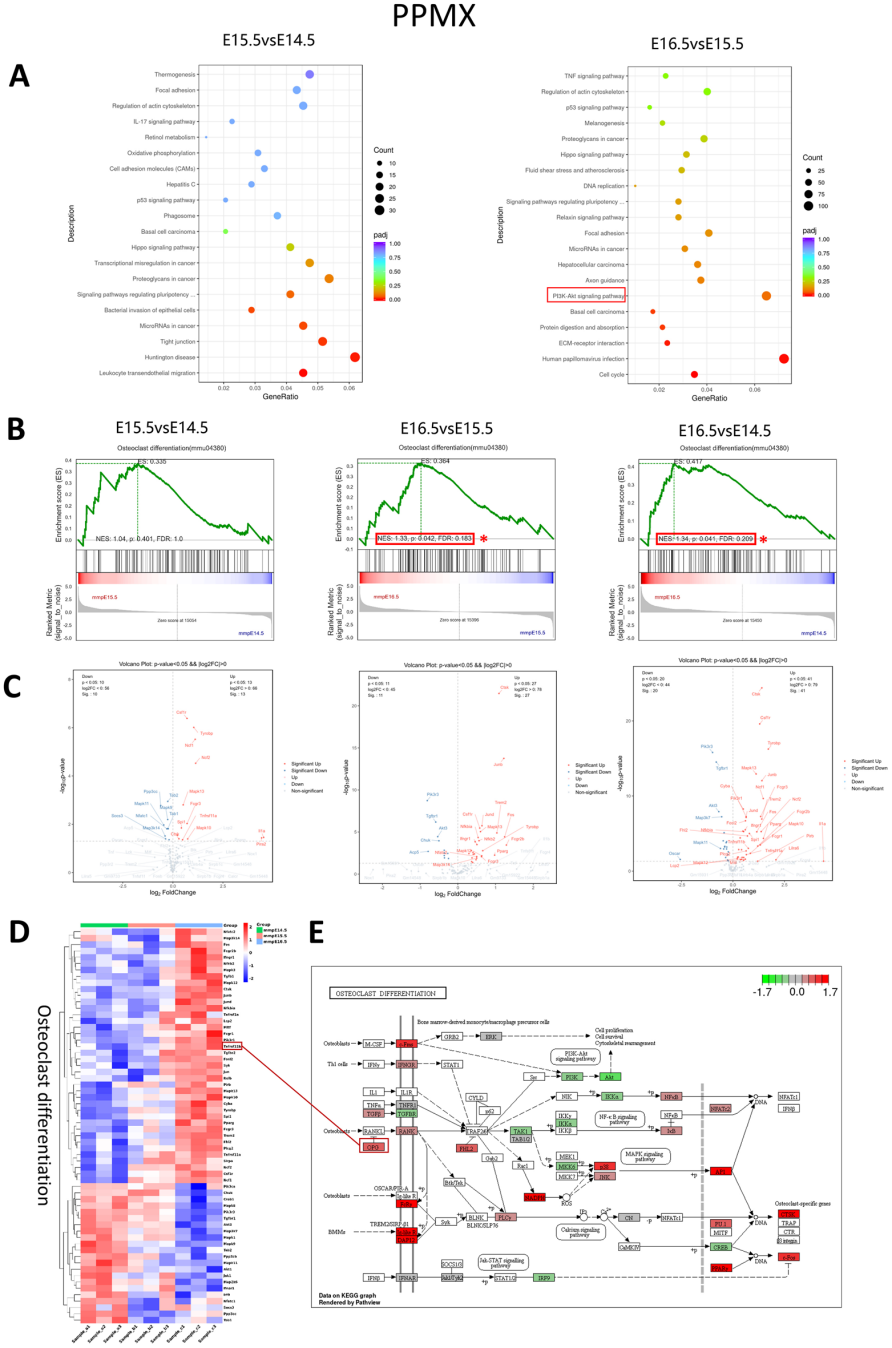
Transcriptome analysis of osteoclast differentiation in PPMX. (**A**) The 20 most enriched KEGG pathways based on DEGs at E15.5 vs. E14.5 and E16.5 vs. E15.5. The x-axis shows the enrichment factor, and the y-axis shows the pathway names. The point size represents the number of genes enriched in a particular pathway. (**B**) GSEA for osteoclast differentiation (mmu04380) on E15.5 vs E14.5, E16.5 vs E15.5 and E16.5 vs E14.5. *Represents significant enrichment of GSEA. (**C**) Volcano plot of OCL differentiation (mmu04380) DEGs on E15.5 vs E14.5, E16.5 vs E15.5 and E16.5 vs E14. 5. Red dots represent upregulated DEGs, and blue dots represent downregulated DEGs. (**D**) Heatmap showing the DEG expression (Z scores) of osteoclast differentiation (mmu04380) at E14.5, E15.5 and E16.5. (**E**) KEGG pathway map of osteoclast differentiation (mmu04380) at E16.5 compared with E14.5. Red boxes represent upregulated DEGs, and green boxes represent downregulated DEGs.

A total of 1521, 4262, and 7546 DEGs were predicted in the pairwise comparisons of E15.5 vs. E14.5, E16.5 vs. E14.5, and E16.5 vs. E14.5, respectively (Supplementary Fig. S1C), by alignment to the KEGG database. In E16.5 vs. E15.5, the PI3K-AKT signalling pathway (MMU04151) was significantly enriched among the top 20 (Fig. 5A) pathways. GSEA of osteoclast differentiation (mmu04380) showed that in E16.5 vs. E15.5, this pathway was significantly enhanced (NES = 1.33, p = 0.042, FDR = 0.183) (Fig. 5B). The volcano map and heatmap of osteoclast differentiation (mmu04380)-related DEGs showed that in E15.5 vs E14.5, E16.5 vs E15.5 and E16.5 vs E14.5, 13, 27, and 41 significantly upregulated DEGs and 10, 11, and 20 significantly downregulated DEGs were found. Among the continuously upregulated DEGs were Csf1r, Tyrobp, Ncf1, Ncf2, Mapk13, Fcgr3, Tnfrsff11a, Spi1, Mapk10, Ctsk, ll1a, Fos, Fcgr2b, and Tnfrsf11b. The consistently downregulated DEGs were Pik3r3, Tgfbr1, Akt3, Mapk3k7, etc. Notably, Tnfrsf11b, a gene encoding OPG protein that inhibits osteoclast differentiation, was gradually upregulated during this process (Fig. 5C–E). KEGG map analysis of DEGs from E16.5 to E14.5 showed that membrane receptors such as c-Fms (Csf1r), IFNGR, RANK, Fcrγ, Ig-likeR and DAP12 were upregulated, while TGFBR was downregulated. Intracellular signals encoding FHL2, PLCy, NADPH, p38, JNK, NF-κB, and IκB were upregulated, and PI3K, AKT, IKKα, TAK1, MKK6, and IRF9 were downregulated. The nuclear signals encoding NFATc2, AP1, PU.1, CTSK, c-Fos and PPARγ were upregulated, while CREB was downregulated (Fig. 5E).

#### The osteoclast differentiation pathway of PPP was upregulated at E15.5–E14.5

**Figure 6:**
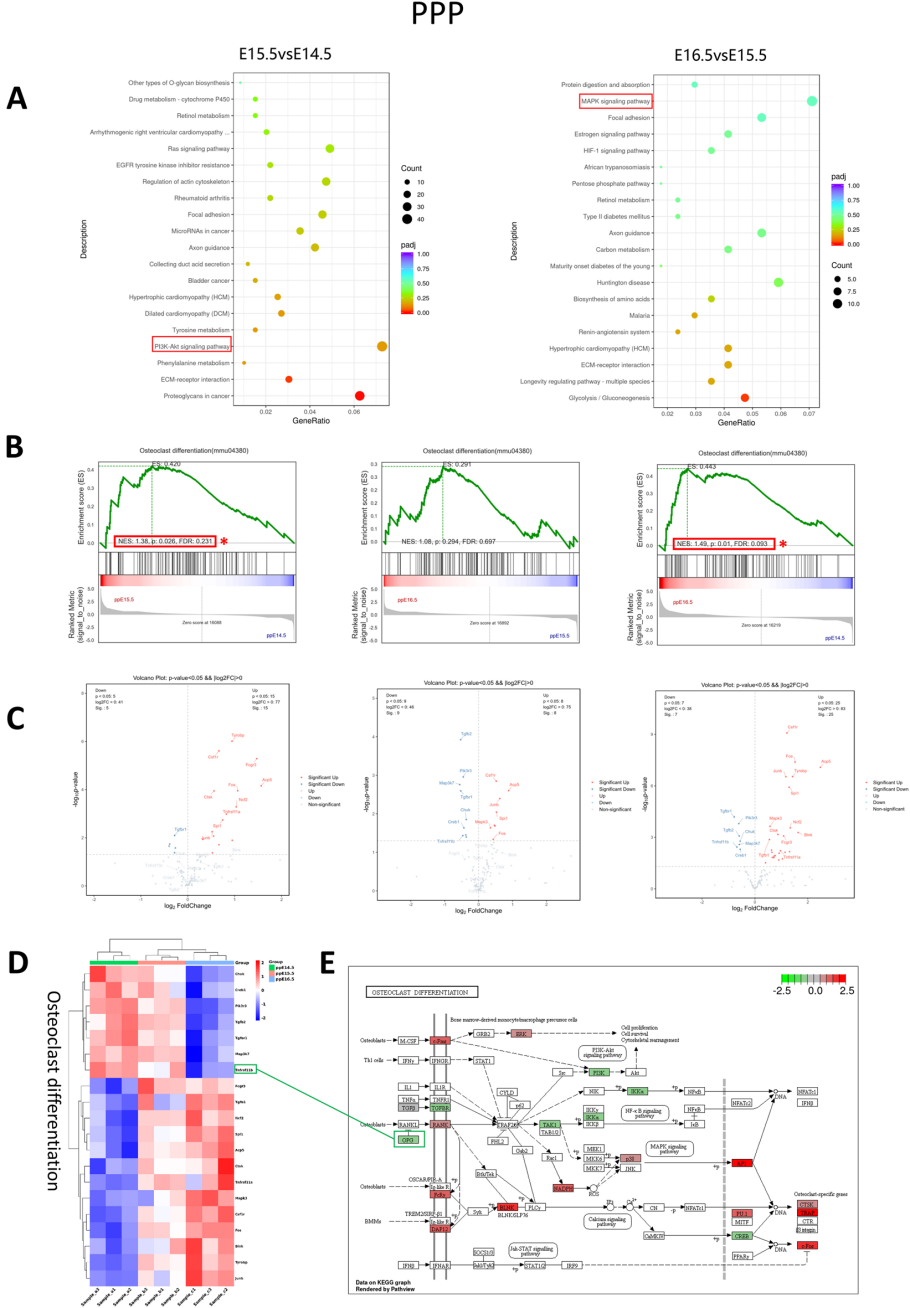
Transcriptome analysis of osteoclast differentiation in PPP. (**A**) The 20 most enriched KEGG pathways based on DEGs at E15.5 vs. E14.5 and E16.5 vs. E15.5. The x-axis shows the enrichment factor, and the y-axis shows the pathway names. The point size represents the number of genes enriched in a particular pathway. (**B**) GSEA for osteoclast differentiation (mmu04380) on E15.5 vs E14.5, E16.5 vs E15.5 and E16.5 vs E14.5. *Represents significant enrichment of GSEA. (**C**) Volcano plot of OCL differentiation (mmu04380)-related DEGs on E15.5 vs E14.5, E16.5 vs E15.5 and E16.5 vs E14. 5. Red dots represent upregulated DEGs, and blue dots represent downregulated DEGs. (**D**) Heatmap shows the DEG expression (Z scores) of osteoclast differentiation (mmu04380) at E14.5, E15.5 and E16.5. (**E**) KEGG pathway map of osteoclast differentiation (mmu04380) at E16.5 compared with E14.5. Red boxes represent upregulated DEGs, and green boxes represent downregulated DEGs.

A total of 1821, 587, and 2561 DEGs were predicted in the pairwise comparisons of E15.5 vs. E14.5, E16.5 vs. E14.5, and E16.5 vs. E14.5, respectively (Supplementary Fig. S1C), by alignment to the KEGG database. In E15.5 vs E14.5 and E16.5 vs E15.5, PI3K-AKT (MMU04151) and the MAPK signalling pathway (MMU04010) were significantly enriched among the top 20 pathways, respectively (Fig. 6A). GSEA of osteoclast differentiation (mmu04380) showed that in E15.5 vs. E14.5, this pathway was significantly enhanced (NES = 1.38, p = 0.026, FDR = 0.231) (Fig. 6B). The volcano map and heatmap of osteoclast differentiation (mmu04380)-related DEGs showed that in E15.5 vs E14.5, E16.5 vs E15.5 and E16.5 vs E14.5, 15, 8, and 25 significantly upregulated DEGs and 5, 9, and 7 significantly downregulated DEGs were found. Among the continuously upregulated DEGs were Csf1r, Tyrobp, Fcgr3, Acp5, Ctsk, Fos, Ncf2, Tnfrsff11a, Spi1, Junb, and Mapk3. The consistently downregulated DEGs were Pik3r3, Tgfbr1, Tgfb2, Chuk, Map3k7, Tnfrsf11b, etc. Notably, Tnfrsf11b was gradually downregulated during this process (Fig. 6C–E). KEGG map analysis of DEGs from E16.5 to E14.5 showed that membrane receptor-encoding mRNA was similar to that of PPMX. Intracellular signals encoding mRNAs were similar to those of PPMX except ERK, AKT, NFκB, IκB, BLNK, PLCγ, IRF9, and FHL2. The nuclear signal-encoding genes were also similar to PPMX except NFATc2, TRAP, and PPARγ (Fig. 6E).

### Quantitative analysis of mRNA expression

**Figure 7:**
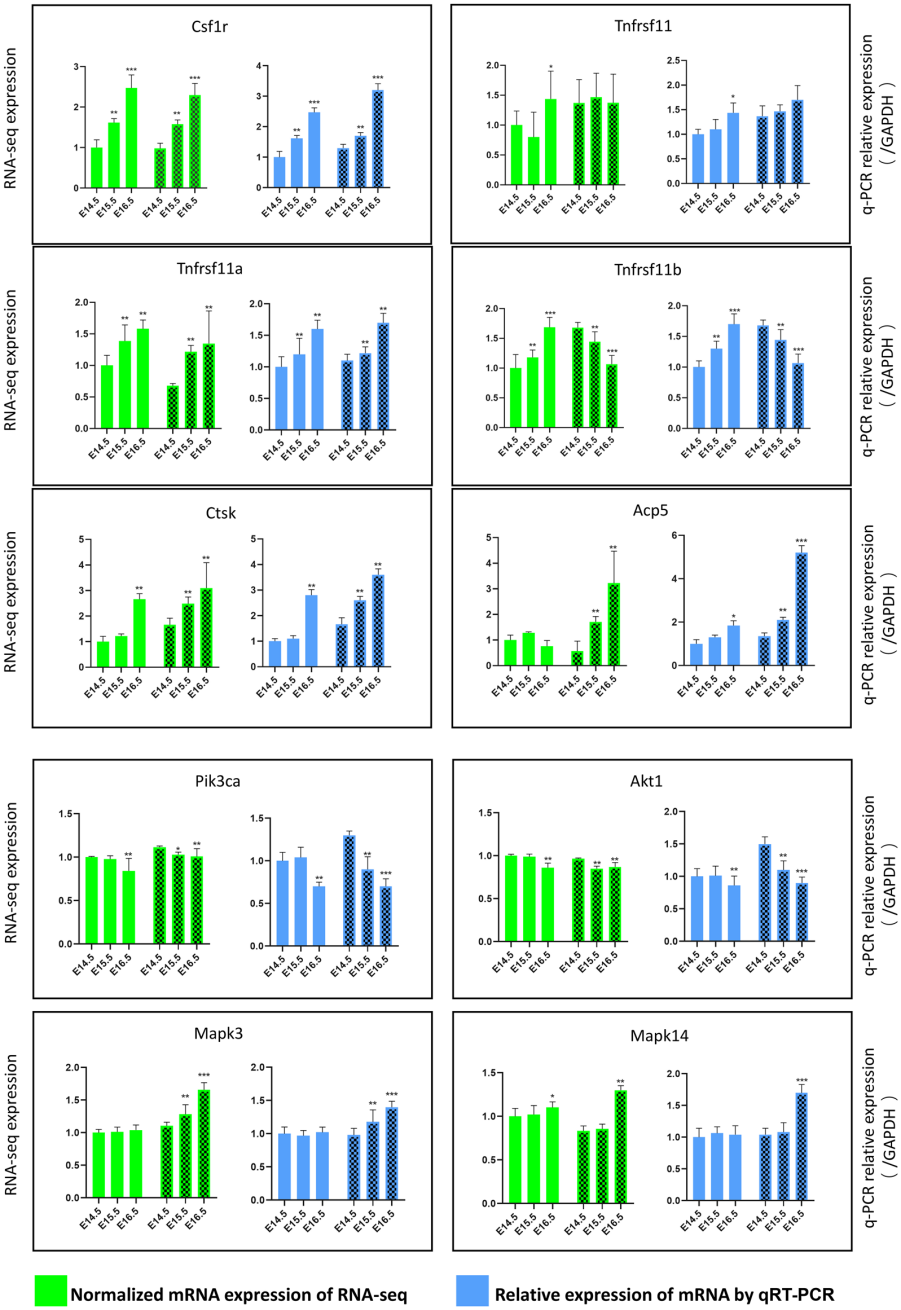
qPCR validation of RNA-seq data. Relative expression levels of mRNA by RNA-seq and qPCR. The upper green panels represent the RNA-seq data, and the blue panels represent the qPCR data. Blank entries represent PPMX, while entries with fill patterns represent PPP *p < 0.05, **p < 0.01, ***p < 0.001.

The validation of expression profiles assessed from the quantity of sequence data captured was performed by qRT-PCR. We selected six genes, which are representative genes corresponding to well-known proteins in osteoclast differentiation. The selected genes were all significant in the differential analysis of the osteoclast gene set in RNA-seq except Tnfrsff11. The expression patterns of the selected mRNAs were consistent with those determined using high-throughput sequencing, which verified that the sequencing data in the present study were reliable and could be subjected to further analysis. The PI3K-AKT pathway genes (Pik3ca, Akt1) and MAPK (Mapk3, Mapk14) were also selected for verified significant enrichment of KEGG for these two pathways. Pik3ca and Akt1, which correspond to the PI3K-AKT signalling pathway, were downregulated, whereas Mapk3 and Mapk14, which correspond to the MAPK signalling pathway, were upregulated at the corresponding time and site of KEGG enrichment. The amplification efficiency of qRT-PCR primers ranged from 93.59 to 104.32% (Fig. 7; Table 1).

## Discussion

Increasing attention has been given to the development of palatal bone as the basis of bone regeneration^3,6–9^. Palate bone ossifies in an intramembranous fashion^10,11^, which involves osteoblast differentiation, ECM assembly and mineralization, vascularization and osteoclast differentiation^30,31^. This study is the first to investigate the differentiation process and gene regulation of palatal OCLs.

**Figure 8:**
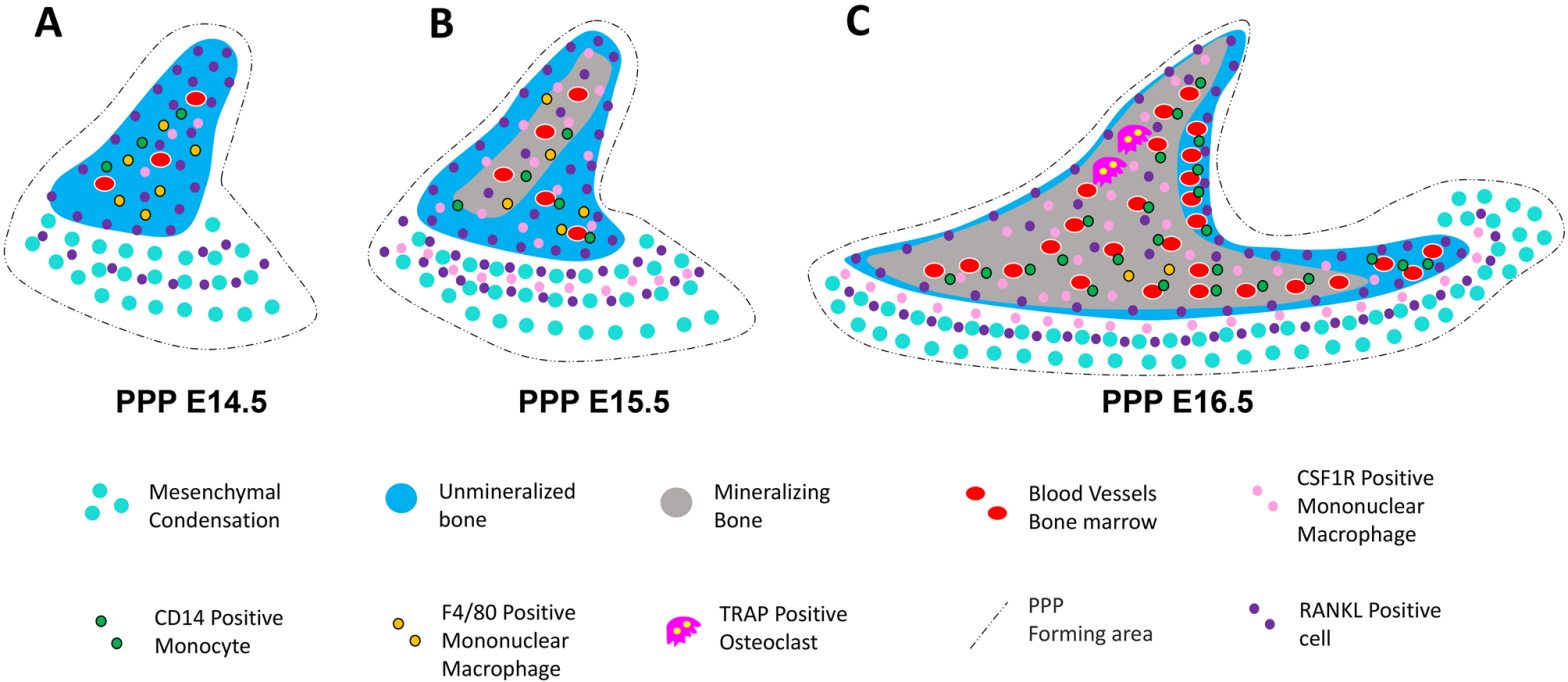
Schematic representation of the spatiotemporal relationship of CD31, CD14, F4/80, CSF1R and RANKL during the osteoclast differentiation process of palatal bone in embryonic mice (PPP). (**A**) At E14.5 in PPP, unmineralized bone was formed at the site of the earliest aggregation of the mesenchyme, and there were CD31-positive vascular endothelial cells in this area, surrounded by CD14-positive monocytes and F4/80 macrophages. CSF1R and RANKL were also expressed in this area. (**B**) At E15.5 in PPP, mineralization was observed at the site of the earliest CD31 vascular expression, which may be due to the entry of mineralized matrix within the blood vessels. CD14-positive monocytes and F4/80-positive macrophages were increased, and RANKL and CSF1R expression was increased. (**C**) At E16.5 in PPP at the earliest sites of blood vessel formation and mineralization, OCLs appear, at which time RANKL and CSF1R expression is strongest. Interestingly, CD14-positive monocytes continued to increase, whereas F4/80 macrophages were reduced in areas of osteoclast formation and expressed in small amounts in other areas.

In studies of intramembranous ossification at the embryonic stage, few studies have focused on the role of OCLs. The current study revealed that TRAP-positive cells appear prior to bone mineralization, suggesting that OCLs contribute to bone matrix maturation during mandible intramembranous ossification^19,32^. In this study, although TRAP-positive OCLs occurred in the palatal bone in a backwards to forwards order, they were consistent with the relationship between mineralization and angiogenesis. After vessel formation, mononuclear macrophages migrate into the palatal bone, and mineralized material is deposited around it. TRAP-positive OCLs appear after the formation of mineralization, in contrast to the aforementioned studies in the embryonic mandible. These OCLs were mainly distributed in the medial part of the bone marrow cavity, suggesting that OCLs may play a role in bone remodelling and bone marrow cavity formation in the palatal bone^33^. The expression of blood vessels increased after the appearance of OCLs, suggesting that OCLs may play a role in inducing angiogenesis^34,35^. These histology-based findings need further validation (Fig. 8).

Transgenic mice are often used to investigate the role of a gene in palate development by altering its expression at specific time points and tissues^36–38^. Therefore, the study of the time and site of OCL differentiation in embryonic palatal bone can be the basis for further research. OCLs are multinucleated cells derived from the monocyte/macrophage lineage, especially from CD14 + monocytes^39,40^. The suppression of macrophage phenotypes, such as F4/80 expression in osteoclast precursors, preceded osteoclastic differentiation. Therefore, macrophages such as F4/80-positive cells may differentiate into osteoclast precursors in vivo^41,42^. Binding of CSF1R to its ligands (typically CSF1) promotes OCL survival and proliferation^21,22^. CSF1R induces RANK expression in multipotent myeloid precursors^43,44^. Binding of RANK by its ligands (RANKL) induces cells of the monocyte-macrophage lineage to differentiate, fuse, and mature into bone-resorbing OCLs^23,45^. In the present study, TRAP-positive OCLs appeared in the palatal bone in a backwards to forwards order (E16.5 at PPP, E17.5 at PPMXP, E18.5 at PPMXA). Before the appearance of TRAP-positive OCLs, CD14-positive monocytes and F4/80-positive macrophages were expressed in the mesenchyme at E14.5 at three sites, indicating that osteoclast precursors had already arrived in the palatal region. CSF1R and RANKL expression was enhanced from 2 days before the appearance of TRAP-positive OCLs (E14.5 at PPP, E15.5 at PPMXP, E16.5 at PPMXA) and was strongest at the time of appearance. These results indicated that OCL differentiation occurred 2 days before the appearance of OCLs and existed in a backwards to forwards order. Interestingly, at the time point and site when TRAP-positive OCLs appeared, CD14-positive monocytes continued to increase, while F4/80-positive macrophages were absent or weakly expressed. This finding indicated that the transformation of monocyte-macrophages into OCLs occurred at this site. This F4/80 expression pattern was similar to that in previous studies^41,42^, indicating that F4/80-positive macrophages have the potential to transform into OCLs of embryonic palatal bone (Fig. 8).

Based on analysis of the time point of OCL differentiation, RNA sequencing was performed on tissues from two sites (PPMX and PPP) at three time points (E14.5, E15.5 and E16.5) to determine the dynamic mRNA expression associated with OCLs in embryonic palatal bone. GSEA showed that osteoclast differentiation (mmu04380) was significantly increased at PPMX and PPP from E16.5–E15.5 and E15.5–E14.5, respectively, which also supported previous findings of palatal osteoclast differentiation from the back to the front 2 days before maturation.

KEGG enrichment analysis showed that the PI3K-AKT signalling pathway was significantly enriched at E16.5–15.5 in PPMX and E15.5–E14.5 in PPP, and the MAPK signalling pathway was significantly enriched at E16.5–E15.5 in PPP. The PI3K-AKT signalling pathway is important for OCL survival, while the MAPK signalling pathway has been shown to promote OCL differentiation^25,27,46^. The verification of the key genes in the corresponding pathways by qRT-PCR suggested that the PI3K-AKT pathway was downregulated one day earlier in PPP than in PPMX, indicating that signals promoting osteoclast survival are weakened one day earlier in PPP than in PPMX. Moreover, the MAPK pathway was subsequently enhanced in the PPP to promote OCL differentiation. This finding illustrates the importance of these two pathways in the differentiation of palatal OCL.

The heatmap shows the dynamic expression of OCL differentiation-related DEGs in the embryonic palatal bone. The volcano plot showed the most DEGs in OCL differentiation. We found that Csf1r, Tnfrsff11a, Ctsk, Fos, Tyrobp, Fcgr3 and Spi1 were significantly upregulated in both PPMX and PPP. Csf1r (encoding colony stimulating factor 1 receptor) and Tnfrsf11a (encoding receptor activator of nuclear factor-κb, RANK) are the two most important receptors that determine OCL differentiation^21,22,43^. Ctsk (encoding cathepsin K) is a marker of mature OCLs. Ctsk deficiency reduces the number of osteoclasts and affects bone morphology^20^. The absence of Fos (encoding transcription factor AP-1 subunit C-Fos) in mice induces substantial osteopetrosis that is caused by the absence of OCLs^44^. Tyrobp [encoding DNAX-activating protein 12 (DAP12)] and Fcgr3 [encoding Fc receptor common gamma subunit, FcRγ)], mice doubly deficient in FcRγ and DAP12 exhibit severe osteopetrosis owing to differentiation blockade of osteoclasts^47^. Pik3r3, Tgfbr1, and Mapk3k7 were significantly downregulated in both PPMX and PPP. Pik3r3 (encoding PI3-kinase regulatory subunit gamma, PI3K) intracellular signalling is associated with osteoclast proliferation and survival^46,48^. Tgfbr1 (encoding transforming growth factor-beta receptor type I, TGFRβ1) plays a potential role in TGF-beta as an important modifier of receptor activators of RANKL-dependent osteoclast activation and osteolysis^49^. Map3k7 (encoding Transforming Growth Factor-Beta-Activated Kinase 1, TAK1) activates the transcription factors AP-1 and NF-κB in response to receptor activator of RANKL stimulation, thus constituting a key regulator of osteoclast differentiation^50^. Interestingly, Tnfrsf11b (encoding osteoprotegin, OPG), which is upregulated in PPMX but downregulated in PPP, is secreted by osteoblasts and acts as a decoy receptor for RANKL, which negatively regulates the process of osteoclast formation. Our previous findings showed that the expression of RUNX2 in palatal bones at E14.5–E16.5, an osteogenic marker, was gradually upregulated in PPMX but downregulated in PPP^9^, suggesting that the different timings of osteoblast differentiation in the anterior and posterior axes of palatal bones may regulate osteoclast differentiation through the RANK-RANKL-OPG axis^23^.

By GSEA, the OCL differentiation pathway of PPP was found to be significantly upregulated 1 day earlier than that of PPMX. Thus, the KEGG pathway maps of OCL differentiation for PPMX and PPP represent the earlier and later stages, respectively. A joint analysis of these two results will allow a more complete understanding of the molecular network of possible osteoclast differentiation in the embryonic palate. OCL differentiation is essentially regulated by three signalling pathways, which are activated by M-CSF-cFms (Csf1r), RANKL-RANK, and the immunoreceptor tyrosine activation motif (ITAM)^51^.

Binding of M-CSF to c-Fms stimulates cell survival mainly by activating extracellular signal-regulated kinase (ERK) through Grb2 and Akt through PI3K^46,48^. From E16.5 to E14.5, CSF1R and ERK were upregulated, while PI3K was downregulated. These results indicated that CSF1R-mediated PI3K and ERK promoted the survival of palatal OCLs at early and late stages of differentiation, respectively.

Following binding of RANKL to RANK, TRAF6 leads to activation of the transcription factor NF-κB. MAPK signalling is also activated via p38 and JNK, which leads to the activation and nuclear translocation of c-Fos, c-Jun, and AP1. Activation of the NF-κB and MAPK signalling pathways induces activation of NFATc1, an essential transcription factor that, together with c-Fos, sets in motion a broad gene expression program that drives osteoclastogenesis and includes genes such as *Acp5 and Ctsk*^27^. In our results, RANK was consistently upregulated, and its mediated NF-κB and MAPK (p38, JNK) signalling pathways were consistently activated, thereby promoting the upregulation of NFATc2 and AP1, potentially inducing the expression of NFATc1. RANKL and NFATc1 were consistently highly expressed at different time points in different sites (data not shown) without upregulation. The coupregulation of NFATc1 and c-Fos also led to upregulation of the osteoclast-specific genes CTSK (PPMX and PPP) and TRAP (PPP).

Multiple immunoglobulin-like receptors associated with ITAM, FcRγ, and DAP12 mediate costimulatory signals of RANK, which activate calcium signalling via phospholipase Cγ (PLCγ). Calcium signalling also activates the calcium/calmodulin activated kinase-cyclic am response element binding protein (CaMK-CREB) pathway^51^. In our results, FcRy and DAP12 were consistently upregulated, along with PLCy, which induces calcium signalling activation, and CAMKIV was consistently highly expressed without temporal differences, whereas CREB was consistently downregulated at this time.

Although we did not aim to identify new genes, the results contribute to the limited knowledge relating mRNAs to specific steps of OCL differentiation in the embryonic palatal bone.

There are some limitations to this study. The inability of the batch RNA-seq used in this study to distinguish osteoclasts from other cells is a limitation of our study, and further study is needed to confirm its physiological importance. However, numerous studies have shown that bulk RNA sequencing is an effective strategy to identify genes and key pathways to provide clues for subsequent studies^2,52,53^. Moreover, studies of the correlation between the transcriptome and proteome in developing mouse osteoclasts can provide us with more valuable and insightful evidence^54^.

## Conclusion

In summary, OCLs may play a role in bone remodelling, bone marrow cavity formation, and angiogenic promotion in embryonic palatal bone. The differentiation process of OCLs began 2 days before maturation, and the order of differentiation and maturation was from the posterior to anterior axis. This study describes the dynamic mRNA and pathways involved in OCL differentiation, which will be important for subsequent studies in normal and abnormal subjects.

### Supplementary Information

Supplementary Figure 1.

Supplementary Legends.

## Data Availability

The RNA-seq datasets generated in this study have been submitted to the Gene Expression Omnibus (GEO) database at the National Center for Biotechnology Information (NCBI) under accession no. GSE240930 (https://www.ncbi.nlm.nih.gov/geo/query/acc.cgi?acc=GSE240930).
