# Supplementary figures and images for: Osteoclast differentiation and dynamic mRNA expression during mice embryonic palatal bone development
Source: Sci Rep. 2023 Sep 13;13:15170. doi: 10.1038/s41598-023-42423-4 (PMC10499879; doi:10.1038/s41598-023-42423-4)

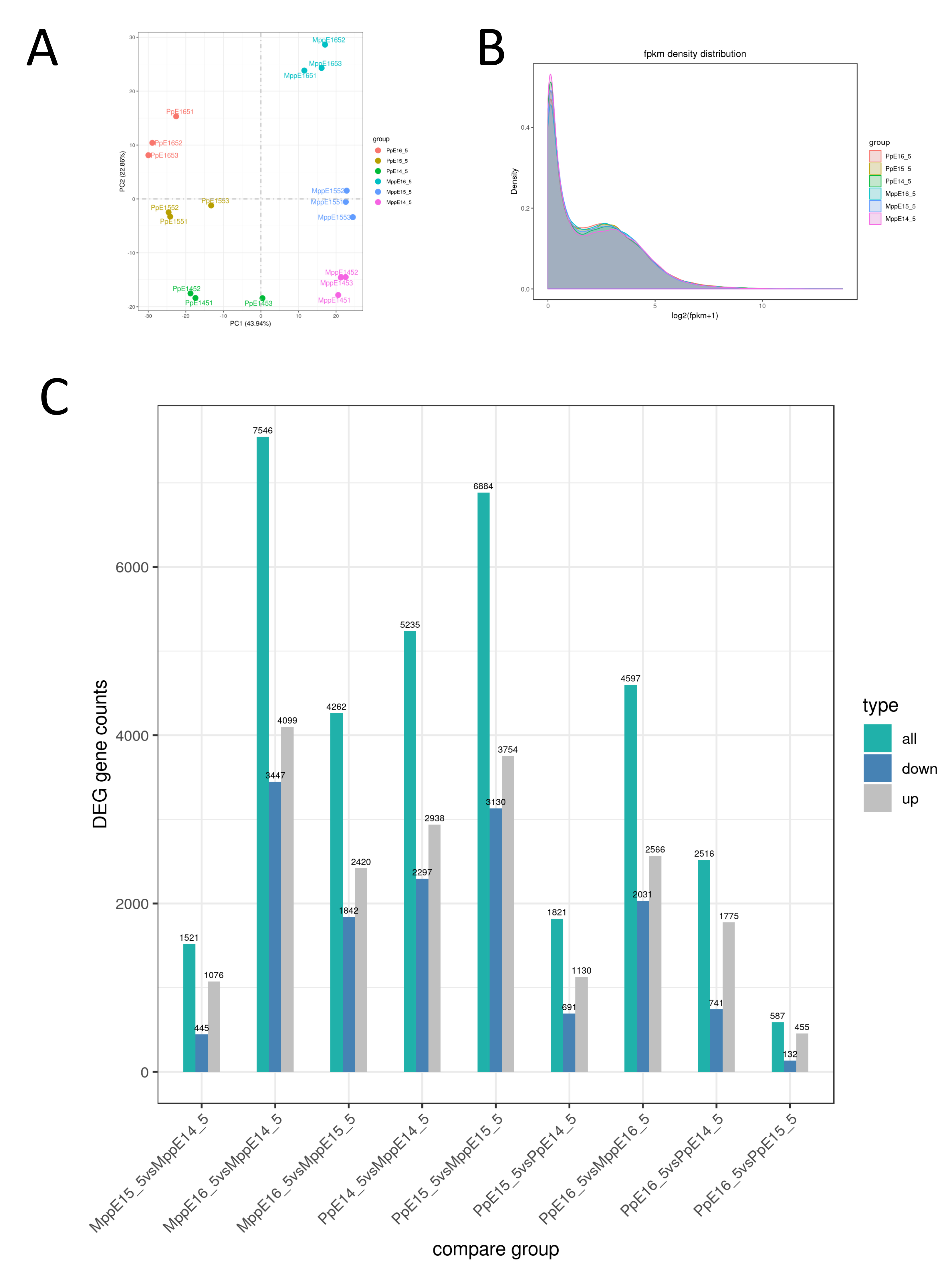

Supplement: Supplementary file 1 — Supplementary Figure 1. [file 41598_2023_42423_MOESM1_ESM.jpg]
