# Supplementary material for: Osteoclast differentiation and dynamic mRNA expression during mice embryonic palatal bone development
Source: Sci Rep. 2023 Sep 13;13:15170. doi: 10.1038/s41598-023-42423-4 (PMC10499879; doi:10.1038/s41598-023-42423-4)
Supplement: Supplementary file 2 — Supplementary Legends. [file 41598_2023_42423_MOESM2_ESM.docx]

**Supplementary Information**

**Supplementary Figure 1**. PCA plot (A), FPKM density distribution (B) and bar graph of differentially expressed genes (C) between PPMX and PPP at E14.5, E15.5 and E16.5.
